# Democratising Artificial Intelligence for Pandemic Preparedness and Global Governance in Latin American and Caribbean Countries

**DOI:** 10.1111/1751-7915.70256

**Published:** 2025-10-22

**Authors:** Ulisses Rocha, Robson Bonidia, Jude Dzevela Kong, Mariana Dauhajre, Claudio Struchiner, Guilherme Goedert, Peter F. Stadler, Danilo Sanches, Troy Day, Marcia C. Castro, John Edmunds, Manuel Colomé‐Hidalgo, Demian Arturo Herrera Morban, Edian F. Franco, Cesar Ugarte‐Gil, Patricia Espinoza‐Lopez, Gabriel Carrasco‐Escobar, André de Carvalho

**Affiliations:** ^1^ Institute of Mathematics and Computer Sciences University of São Paulo São Carlos Brazil; ^2^ Department of Computational Biology and Chemistry Helmholtz Centre for Environmental Research‐UFZ GmbH Leipzig Germany; ^3^ Department of Computer Science Federal University of Technology‐Paraná (UTFPR) Cornélio Procópio Brazil; ^4^ Department of Mathematics and Statistics York University Toronto Ontario Canada; ^5^ Centro de Investigación en Salud Dr. Hugo Mendoza Hospital Pediátrico Dr. Hugo Mendoza Santo Domingo Dominican Republic; ^6^ Escola de Matemática Aplicada Fundação Getúlio Vargas Rio de Janeiro Brazil; ^7^ Department of Computer Science and Interdisciplinary Center of Bioinformatics University of Leipzig Leipzig Germany; ^8^ Department of Mathematics and Statistics Queen's University Kingston Ontario Canada; ^9^ Department of Global Health and Population Harvard T.H. Chan School of Public Health Boston Massachusetts USA; ^10^ London School of Hygiene and Tropical Medicine London UK; ^11^ Instituto de Investigación en Salud Universidad Autónoma de Santo Domingo (UASD) Santo Domingo Dominican Republic; ^12^ Centro de Investigación UTESA Universidad Tecnologica de Santiago (UTESA) Santiago de los Caballeros Dominican Republic; ^13^ School of Basic and Environmental Sciences Instituto Tecnológico de Santo Domingo (INTEC) Santo Domingo Dominican Republic; ^14^ Department of Epidemiology The University of Texas Medical Branch Galveston Texas USA

**Keywords:** AI governance, AI in pandemic response, democratisation of AI, equitable public health systems, global south initiatives, responsible AI use

## Abstract

Infectious diseases continue to pose a significant global health challenge, necessitating innovative approaches for predicting outbreaks, detecting variants, conducting contact tracing, discovering new drugs and managing misinformation. Artificial intelligence (AI) has significantly supported work in these areas, particularly during the COVID‐19 pandemic. However, the benefits of AI must be equitably distributed, and its use must be responsible and inclusive. As various nations implement AI regulations, the global nature of AI necessitates international collaboration to establish ethical guidelines and governance frameworks. In response to these needs, the Global South AI for Pandemic & Epidemic Preparedness & Response Network (AI4PEP) is leading a multinational effort across 16 countries to strengthen public health systems through responsible, Southern‐led AI solutions. This opinion piece highlights AI4PEP's initiatives in Latin America and the Caribbean (LAC), examining the region's AI governance models and the challenges they present. By lowering barriers to AI adoption and fostering equitable access to AI‐driven public health innovations, our network empowers researchers, healthcare professionals and policymakers in LAC to harness AI for infectious disease preparedness and response, ultimately improving health outcomes in low‐ and middle‐income countries.

## Graphical abstract

This piece highlights how the AI4PEP network empowers Latin American and Caribbean countries to adopt responsible, locally driven AI for pandemic preparedness, addressing global governance challenges while promoting equitable access, innovation and inclusion in public health.

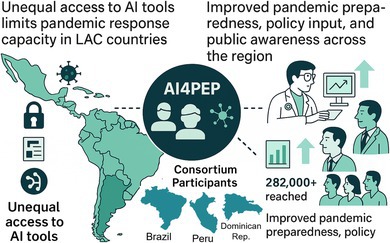

## 1 Introduction

Lessons learned from the COVID‐19 pandemic outbreak highlight the need to improve our preparedness for similar events (Alakija 2023). This pandemic has shown us that no single person, community or country is isolated, and that suffering and a lack of support affecting individuals, regardless of their location, affect us all. Moreover, we must recognise talent, wherever and whoever they are, to join and collaborate in the search for solutions to the world's challenges.

In this effort, Artificial Intelligence (AI) emerges as a valuable tool for reducing the impacts of pandemics (Syrowatka et al. 2021). In an era where AI is present in various processes that impact society, it is essential to ensure that its contributions are distributed equitably (Seger et al. 2023). Moreover, the democratisation of AI must empower each individual, community or society to contribute proportionally to their aptitude, availability and commitment, thereby increasing the implementation of AI in practical applications (Ahmed and Wahed 2020).

With AI advances, concerns and contributions, the world has received both positive and negative signals (i.e., ethically, technologically and societally) regarding the future of a world where AI is firmly present (Colavizza et al. 2021). Advances in all knowledge domains demonstrate how AI can not only accelerate scientific discoveries and the design of innovative solutions, many of which improve people's health, but also be one of the most valuable tools for enhancing the quality of life on earth (Gupta and Degbelo 2023). On the other hand, recent articles, talks and interviews address the dangers of developing and using AI (Colavizza et al. 2021; Rajpurkar et al. 2022; Gerke 2024).

Furthermore, the development of AI has been concentrated in countries with a long focus on technological advancements, overlooking the unique needs and challenges faced by less developed regions, such as the Global South (The ‘AI Divide’ Between the Global North and Global South 2023). A study assessing 181 countries worldwide reveals that many nations in the Global South rank among those with the lowest AI readiness scores (2024 Government AI Readiness Index n.d.). The study emphasises that, without a supportive environment, these disparities could deepen global inequality in access to and development of AI (Okolo et al. 2022).

Consequently, law‐making bodies in many countries and regions are passing new regulations regarding the development and use of AI to reduce or avoid risks associated with this technology (Anderljung et al. 2023; Hacker et al. 2023). These regulations may prevent biased AI systems, ensure fairness and improve privacy protection. Nevertheless, AI has no borders. Potential risks that may not be adequately addressed in countries with permissive or absent AI regulation include practices such as training biased algorithms or exploiting personal data without consent. Even when some nations adopt stricter frameworks, the global circulation of AI systems through the internet means that weak regulation elsewhere can still expose users in well‐regulated environments to these risks. Recent articles have defended the idea that AI regulation should not be the responsibility or work of a single country or region.

Therefore, a global board should indicate the best actions to create legislation regulating AI, similar to the Intergovernmental Panel on Climate Change (IPCC), an intergovernmental body of the United Nations (Ho et al. 2023; Meskó and Topol 2023). The democratisation of AI aims at its ethical and responsible expansion, encompassing its development, use and governance (Ahmed and Wahed 2020; Himmelreich 2023). These aims require equal opportunities worldwide. Furthermore, we should plan to share knowledge and training involving AI to benefit all regions of the globe (Himmelreich 2023).

Considering these issues, York University (Toronto, Canada) was selected in a Canadian national bid for a grant from the International Development Research Centre (IDRC) (AI for Global Health Investment) (Artificial Intelligence for Global Health | IDRC—International Development Research Centre 2024) to support countries in the global south to fight infectious diseases using AI, the Global South AI for Pandemic & Epidemic Preparedness & Response Network (AI4PEP) (Artificial Intelligence for Global Health | IDRC—International Development Research Centre 2024). This network comprises 16 projects from 16 countries in the global south. This opinion introduces our branches in Latin American and Caribbean (LAC) countries and discusses AI governance in the LAC region.

The Brazilian branch of the network, AutoAI‐Pandemics, investigates and designs automated machine learning (ML) tools to democratise healthcare professionals' use of AI. These tools support (1) automated epidemiological analysis for designing interventions, (2) automated bioinformatics analysis and (3) fighting misinformation/disinformation. AutoAI‐Pandemics hopes to democratise access to data science and ML techniques by allowing non‐experts (e.g., biologists, physicians and epidemiologists) to use AI in their research and development.

The Dominican branch, AI4EWARS, uses AI to model the spread of Aedes‐borne diseases in the country. It comprises multidisciplinary and experienced health researchers, including entomologists, mathematicians and medical doctors. They plan to design a predictive model of Aedes‐borne viruses in the Dominican Republic that provides early warnings of outbreaks and streamlines Public Health responses.

The AI4PEP hub in Peru enhances an existing database of forced cough sounds to further train pilot AI algorithms for classifying various respiratory infectious diseases in patients with respiratory symptoms. The project aims to evaluate the utility of longitudinal cough monitoring in indexing patients and their household contacts, and to integrate AI‐based tools to improve respiratory infection surveillance, access and equity within the Peruvian healthcare system.

Our network in LAC has the potential to reduce the expertise barrier required to engage with ML pipelines, making AI tools and applications more accessible to researchers fighting infectious diseases, particularly in low‐ and middle‐income countries. This initiative enables biologists, physicians, epidemiologists and other stakeholders to apply these techniques widely, thereby improving the health and well‐being of their communities. Ultimately, by providing a simplified approach to conducting complex AI analyses, we encourage the broader inclusion of researchers from diverse backgrounds and resources, thereby strengthening the global commitment to science and health.

## 2 Challenges and Inequalities in LAC

AI solutions have been proposed in several domains (e.g., healthcare, finance, education and agriculture). In October 2022, the Food and Drug Administration (FDA) reported 521 AI and ML‐enabled medical devices (Joshi et al. 2024). Furthermore, the COVID‐19 pandemic demonstrated the potential of ML techniques to minimise the effects of a pandemic, such as predicting deaths, contact tracing, diagnosis, treatments and others (Luengo‐Oroz et al. 2020; Comito and Pizzuti 2022).

Nevertheless, many ML algorithms and AI models have a black‐box nature (i.e., AI decisions are not comprehensible on a human level) (Rudin 2019; Babic et al. 2021), which may reduce trust, accountability and acceptance of AI (Hutter et al. 2019). Another concern is that ML models can inadvertently reflect and amplify hidden social biases present in the training data, leading to unfair, harmful or prejudiced decisions. These biases often arise from systemic issues or human oversight in data collection and curation, rather than being inherent flaws of the technology itself. Some examples of these problems have been reported in various fields.

In 2009, genome‐wide association studies revealed that more than 96% of participants were of European descent (Popejoy and Fullerton 2016), thereby failing to encompass the full range of racial and geographic diversity, which can limit the applicability of the findings to diverse populations. A study regarding bias in AI highlighted sex and gender biases in AI applications for biomedicine and healthcare, emphasising the need for more inclusive data representation (Cirillo et al. 2020). Similarly, in dermatology, medical imaging and diabetes management, studies have identified a lack of racial diversity in training datasets, raising concerns about potential health disparities (Adamson and Smith 2018; Pham et al. 2021; Ricci Lara et al. 2022).

Consequently, initiatives have discussed concerns regarding the ethical, fair, reliable, sustainable, transparent and reproducible AI (Shang et al. 2019). Thus, in our pursuit of responsible solutions, our network adheres to guidelines proposed in the literature, such as those on developing and using AI responsibly (Dignum 2019), AI for all (Ramos 2021) and the Guidelines for Trustworthy AI (Zhang and Zhang 2023). We also adopt and recommend the principles of Data‐Centric AI (Whang et al. 2023), which puts data at the heart of an AI system development process, and the guidelines to improve the Findability, Accessibility, Interoperability and Reuse of data known as FAIR data principles (Wilkinson et al. 2016).

## 3 Comparative AI Governance Models in LAC

According to a report by the OECD (2022), AI capacities in LAC countries vary widely. Countries like Trinidad and Tobago, Venezuela and Bolivia have yet to achieve significant public sector development. Uruguay and Colombia are regional leaders in AI strategy research and development. These countries are followed by Peru, Chile, Brazil, Costa Rica and Argentina, which have drafted or enacted legislation about AI. In addition, Mexico was the first LAC nation to create a national strategy for AI, as shown in Table 1. Lastly, Paraguay, Panama, Jamaica, Ecuador, the Dominican Republic and Barbados are beginning to integrate public sector strategies for AI.

**TABLE 1:** Overview of AI laws, strategies and definitions in different Latin American countries.

Year	Country	AI definition	Comparison and contrast
2012–2013	Colombia	No explicit definition	Colombia has a personal data protection law and an International AI Council for Colombia
2018	Mexico	No explicit definition	Mexico presented the first national AI strategy in the Latin American and Caribbean region in March 2018. The strategy focuses on ethics, governance, investment and innovation
2019	Brazil	The Brazilian AI Strategy defines AI as ‘a set of techniques that enable machines to perform tasks that, if performed by humans, would require intelligence’	The strategy focuses on AI research and development, as well as ethics and governance
2021	Chile	No explicit definition	Chile has a national AI policy focusing on ethics, governance and innovation
2021	Peru	No explicit definition	Peru has a national AI policy focusing on ethics, governance and innovation

According to the same OECD Report, Annex A (OECD 2022), we describe the main features and points regarding LAC nations' AI strategies, including:

- *Uruguay* (*AI Strategy for the Digital Government*) focuses on applying AI to public administration, emphasising capacity building, responsible use and promoting digital citizenship.
- *Peru* (*National Strategy for Artificial Intelligence*) addresses infrastructure, ethics, training and new economic models, with a commitment to renew and update the strategy every 2 years after 2026.
- *Mexico* (*IA‐MX 2018*) has an AI subcommittee to coordinate government, academia and industry, promoting cross‐sectoral collaboration and participation in international technology working groups.
- *Colombia* (*Digital Transformation and AI National Policy*) integrates AI into the modernisation of government services and public sector innovation, highlighting efficiency and citizen engagement.
- *Chile* (*AI Action Plan and AI National Policy*) emphasises the integration of AI into sustainable development, human well‐being and regulatory frameworks, linking AI to broader societal goals.
- *Brazil* (*Brazilian Artificial Intelligence Strategy*) provides one of the most comprehensive frameworks, combining regulation, public‐service applications, legislation, ethics and international positioning.
- *Argentina* (*AI National Plan*) aims to enhance state efficiency and citizen services through the use of AI, with a particular focus on aligning technology adoption with social needs.
- *Uruguay* (*Agenda Uruguay Digital 2020*) converges its national AI strategy by combining responsible and equitable use of AI, with explicit protections for user privacy (Veronese and Lemos 2021a, 2021b).
- *Costa Rica* gained attention in 2022 when lawmakers drafted an AI regulation bill using ChatGPT. The proposal highlighted the need for legislation governing the use and development of AI (Editorial Team at World Litigation Forum 2023).
- *The Dominican Republic* has articulated a national stance on AI but has not yet defined a concrete mechanism or roadmap for enforcement.

The OECD report provides valuable guidance for LAC governments on maximising AI's positive impacts and minimising the negative ones. By following these recommendations, governments can help to ensure that AI is used for the benefit of all. Overall, the strategies converge on several common priorities, including ethics, governance and the use of AI in public services. At the same time, they differ in emphasis: Chile and Peru highlight sustainability, while Brazil stresses regulatory comprehensiveness and international positioning. Mexico, Colombia, and Argentina place a stronger focus on citizen engagement and aligning AI adoption with social needs.

## 4 Democratising AI Knowledge in LAC

The growing interest in AI has reached several areas and, in many cases, has directly impacted people's lives, particularly in healthcare. However, scaling the implementation of AI innovations requires adaptation and acceptance, which are often challenging. Moreover, in the Global South, specifically in the LAC region, we face significant challenges, including limited accessibility to, as well as funding for, AI resources and a knowledge gap. The complexity of AI techniques and the skills required to apply them create a barrier, restricting the potential for innovative research. Another critical issue is the necessary infrastructure to implement these solutions, as many institutions still struggle with inadequate access to high‐quality internet and computational resources.

Therefore, understanding which factors influence the adoption of new ideas is crucial. Thus, our network proposes to use Rogers' Diffusion of Innovation Theory, which aims to explain how new ideas or innovations can be adopted, such as (1) relative advantage, (2) compatibility, (3) complexity, (4) experimentability and (5) observability (Rogers 1995). This theory can be complemented by other measures for innovation adoption, such as cost‐efficacy, feasibility, perceived evidence, innovation fit with users' norms, relevance and ease (Chor et al. 2015).

**FIGURE 1:**
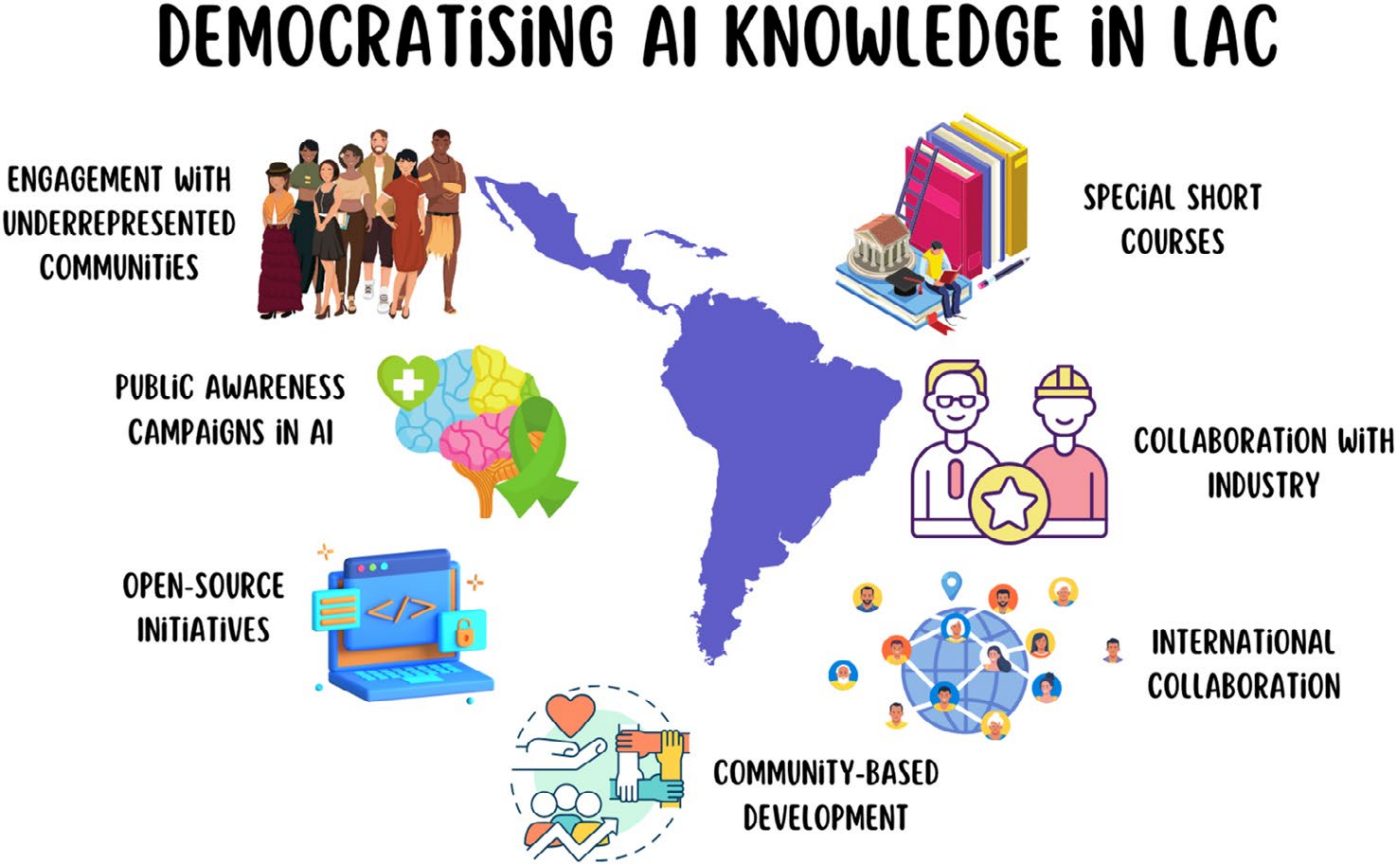
Democratising AI knowledge in Latin America and Caribbean countries.

To democratise AI knowledge, we maximise the dissemination of results and products among the target communities where our motivating issues arise, as well as the training of human resources. Therefore, our network proposes several strategies (Figure 1), such as (1) engagement with underrepresented communities; (2) offer of short AI‐related courses; (3) public awareness campaigns in AI; (4) collaboration with industry; (5) international collaboration; (6) open‐source initiatives and (7) community‐based development (Table 2).

**TABLE 2:** Our key strategies for inclusive AI development in LAC.

Strategy	Implementation examples
Engagement with underrepresented communities and community‐based development	Our educational project, called InteliGente, is dedicated to promoting equity in AI development by educating and empowering individuals to create socially impactful solutions for underserved communities
Special short courses	Several courses and competitions have been organised by the network, such as: (1) I Advanced Summer School on Responsible AutoML; (2) AI4GHI Challenge 2024—Student Summit; AI4PEP Colloquia; (3) Podcast Series and (4) AI4PEP Lecture Series
Public awareness campaigns in AI	Numerous materials have been made available, such as: (1) Data Science—Fundamentals and Applications; (2) InteliGenteCards—Deciphering AI; (3) AI4PEP Newsletter; (4) Policy Perspectives; (5) Our researchers participated in the commission responsible for creating an international report on AI safety; (6) Participants in the Artificial Intelligence Plan of the Brazilian Computer Society
International collaboration	We are an international network spanning 16 countries in the Global South, holding biweekly and monthly meetings to exchange knowledge and share ongoing work. Among the countries are Brazil, Peru, Dominican Republic, Cameroon, Ethiopia, Ghana, Senegal, South Africa, Indonesia, Malaysia, Philippines, Lebanon, Morocco, Tunisia, West Bank
Open‐source initiatives	Our network has developed several open‐source initiatives, including BioAutoML, Dominique, BioPrediction, MuDoGeR, BioDeepFuse, MathFeature, InteliGenteCards, among others

Considering this, our AI4PEP‐LAC initiative has already made a tangible impact through projects like InteliGente, which empowers students to apply AI for social good and has been recognised by the Regional Fund for Digital Innovation in Latin America and the Caribbean (FRIDA) as one of the most transformative initiatives in the LAC region. Additionally, AutoAI‐Pandemics has played a crucial role in leveraging AI to enhance pandemic preparedness by distributing various open‐source solutions that enable non‐specialists to harness the power of AI, such as BioAutoML (Bonidia et al. 2022), BioPrediction (Florentino et al. 2024) and Dominique (Information Integrity Assistant).

Beyond these projects, our contributions extend to the policy sphere, with participation in the development of documents for public policies on AI governance and ethical implementation, such as (1) International AI Safety Report (International AI Safety Report 2025 2025), (2) Strengthening health systems through responsible AI: an emergent research landscape (Sinha 2025), (3) Fostering responsible innovation (Artificial Intelligence for Global Health | IDRC—International Development Research Centre 2024) and (4) Brazilian Artificial Intelligence Plan 2024–2028 (PBIA n.d.). These results illustrate how our multifaceted approach is driving meaningful change across education, research and policymaking in the LAC region.

Furthermore, our network organises and supports academic meetings to strengthen the collaboration of our multinational team and bridge it to local groups in LAC. In these events, we provide novel training sessions to environmental and healthcare professionals and students (e.g., tools developed for intervention design and combating misinformation) (AI4PEP at the UNGA79 Science Summit—AI4PEP n.d.). We also encourage new partnerships addressing key challenges explicitly faced by AI initiatives in the Global South and make special efforts to finance the participation of promising students from other developing countries, providing them with novel training opportunities and possibilities for continued education. For example, we organised a student summit (AI4GHI Challenge 2024—Student Summit—AI4PEP n.d.). In this summit, we proposed challenges that engaged 140 students from 21 countries, bringing together expertise from health sciences, computer science, environmental science and related fields. The participants were tasked with addressing five pressing themes: from predictive analytics for disease outbreaks to the intersection of climate change and mental health. Despite their varied disciplines, a common thread bound them: the drive to make a tangible impact on global health systems.

In our efforts to promote the democratisation of AI across LAC countries, our consortium has made remarkable strides in visibility and engagement. Our direct interactions have provided over 100,000 people with access to various resources, including articles, educational programs and engaging events, demonstrating a significant level of interest and participation in our initiatives. Beyond these direct impacts, the ripple effect of our work has reached an astounding 182,000 individuals through indirect channels. This broad influence can largely be attributed to our robust media coverage and social media outreach. We estimate that approximately 32,000 people have been touched through awards and recognitions, amplifying our message and reaching thousands in their respective communities. Meanwhile, our presence in the media has been equally impactful, with major news outlets alone giving us a voice that resonates with around 150,000 individuals.

When we combine these figures, the total estimated impact of our consortium exceeds 282,000 individuals. This estimate is a testament to our advocacy and reflects a careful and conservative approach to measuring our reach. We ensure that our claims are based on solid data from website analytics, social media metrics, award announcements and media reporting. This extensive outreach illustrates the numbers and the growing awareness and engagement with the democratisation of AI in our region. It is a call to action, showing that we can continue to inspire and empower communities throughout LAC with collective effort. As we approach the conclusion of our work, these figures remind us of the potential we have to change the narrative around AI and its accessibility.

## 5 Conclusions

Harnessing AI's potential in LAC requires not only technological advancements but also ethical and responsible issues, inclusive policies and international collaboration. Our network's efforts to democratise AI knowledge and tools aim to bridge existing knowledge gaps, empowering healthcare professionals, researchers and policymakers to leverage AI for public health and beyond. By fostering accessibility, responsible innovation and global cooperation, we can ensure AI contributes to a more equitable and prepared world where the LAC region actively shapes the future of AI governance and its ethical application.

## Author Contributions

**Ulisses Rocha:** conceptualization, funding acquisition, writing – original draft, writing – review and editing, supervision, resources. **Robson Bonidia:** conceptualization, writing – original draft, writing – review and editing, supervision. **Jude Dzevela Kong:** conceptualization, writing – original draft, writing – review and editing, funding acquisition, supervision, resources, project administration. **Mariana Dauhajre:** writing – original draft, writing – review and editing. **Claudio Struchiner:** writing – original draft, writing – review and editing. **Guilherme Goedert:** writing – original draft, writing – review and editing. **Peter F. Stadler:** writing – original draft, writing – review and editing. **Danilo Sanches:** writing – original draft, writing – review and editing. **Troy Day:** writing – original draft, writing – review and editing. **Marcia C. Castro:** writing – original draft, writing – review and editing. **John Edmunds:** writing – original draft, writing – review and editing. **Manuel Colomé‐Hidalgo:** writing – original draft, writing – review and editing. **Demian Arturo Herrera Morban:** writing – original draft, writing – review and editing. **Edian F. Franco:** writing – original draft, writing – review and editing. **Cesar Ugarte‐Gil:** writing – original draft, writing – review and editing. **Patricia Espinoza‐Lopez:** funding acquisition, visualization. **Gabriel Carrasco‐Escobar:** writing – original draft, writing – review and editing. **André de Carvalho:** writing – original draft, writing – review and editing, conceptualization, funding acquisition, supervision, resources, project administration.

## Ethics Statement

The authors have nothing to report.

## Consent

The authors have nothing to report.

## Conflicts of Interest

The authors declare no conflicts of interest.

## Data Availability

Data sharing not applicable to this article as no datasets were generated or analysed during the current study.
